# Clinical Feasibility of Deep Learning-Based Image Reconstruction on Coronary Computed Tomography Angiography

**DOI:** 10.3390/jcm12103501

**Published:** 2023-05-16

**Authors:** Seul Ah Koo, Yunsub Jung, Kyoung A Um, Tae Hoon Kim, Ji Young Kim, Chul Hwan Park

**Affiliations:** 1 Department of Radiology and The Research Institute of Radiological Science, Gangnam Severance Hospital, Yonsei University College of Medicine, Seoul 06273, Republic of Korea; 2 Research Team, GE Healthcare Korea, Seoul 04637, Republic of Korea

**Keywords:** coronary computed tomographic angiography, deep learning-based image reconstruction, image quality

## Abstract

This study evaluated the feasibility of deep-learning-based image reconstruction (DLIR) on coronary computed tomography angiography (CCTA). By using a 20 cm water phantom, the noise reduction ratio and noise power spectrum were evaluated according to the different reconstruction methods. Then 46 patients who underwent CCTA were retrospectively enrolled. CCTA was performed using the 16 cm coverage axial volume scan technique. All CT images were reconstructed using filtered back projection (FBP); three model-based iterative reconstructions (MBIR) of 40%, 60%, and 80%; and three DLIR algorithms: low (L), medium (M), and high (H). Quantitative and qualitative image qualities of CCTA were compared according to the reconstruction methods. In the phantom study, the noise reduction ratios of MBIR-40%, MBIR-60%, MBIR-80%, DLIR-L, DLIR-M, and DLIR-H were 26.7 ± 0.2%, 39.5 ± 0.5%, 51.7 ± 0.4%, 33.1 ± 0.8%, 43.2 ± 0.8%, and 53.5 ± 0.1%, respectively. The pattern of the noise power spectrum of the DLIR images was more similar to FBP images than MBIR images. In a CCTA study, CCTA yielded a significantly lower noise index with DLIR-H reconstruction than with the other reconstruction methods. DLIR-H showed a higher SNR and CNR than MBIR (*p* < 0.05). The qualitative image quality of CCTA with DLIR-H was significantly higher than that of MBIR-80% or FBP. The DLIR algorithm was feasible and yielded a better image quality than the FBP or MBIR algorithms on CCTA.

## 1. Introduction

Coronary computed tomography angiography (CCTA) is a non-invasive test for diagnosing coronary artery disease (CAD) [1,2,3]. To improve the accuracy of diagnosis and prognosis prediction, a good quality image is mandatory, which usually requires increasing the amount of radiation exposure or contrast agent [4,5,6]. There have been various efforts to improve CCTA image quality while reducing radiation exposure and contrast agents, and one method is to develop improved reconstruction technology.

Filtered-back-projection (FBP), which has been used for the longest time, is an efficient method that is reconstructed in near real-time while scanning the target [7,8]. However, to obtain a good-quality image, the radiation dose could be a drawback. Iterative reconstruction (IR) has been developed to obtain a good image with less radiation and has been commonly used in recent years [9,10,11]. However, the downside of IR is that the image texture is artificial and unnatural [12].

A new reconstruction algorithm is being developed to compensate for the shortcomings of FBP and IR, one of which is the deep-learning-based iterative reconstruction (DLIR). It is programmed to perform reconstruction through a training process that constantly compares the FBP reconstruction of the high-dose sinogram with that of the low-dose sinogram, using a deep convolutional neural network. Therefore, DLIR could improve the image quality of CCTA without additional radiation exposure or save the radiation dose maintaining the image quality. In addition, DLIR enables the overcoming of the unnaturalness of IR by making the image texture close to that of FBP. However, only a few studies have been conducted on the use of DLIR in clinical CCTA.

This study aimed to determine whether DLIR can be applied in a clinical setting by comparing images reconstructed by FBP, model-based iterative reconstruction (MBIR), and DLIR.

## 2. Materials and Methods

### 2.1. Phantom Study

#### 2.1.1. Noise

A 20 cm water phantom was scanned with a 256-detector-row CT scanner (Revolution CT; GE Healthcare, Waukesha, WI, USA) using the following parameters: 100 kVp tube voltage, a noise index of 37.8, and a 0.625 mm slice thickness. Seven sets of images were reconstructed using FBP, MBIR (ASiR-V), and DLIR (TrueFidelity™): FBP, MBIR-40%, MBIR-60%, MBIR-80%, DLIR-Low (L), DLIR-Medium (M), and DLIR-High (H). Within the phantom images, four 4 cm × 4 cm regions of interest (ROIs) were drawn, and the mean noise was compared according to the reconstruction methods.

#### 2.1.2. Noise Power Spectrum

Three sets of images of a 20 cm water phantom were obtained as follows: (1) high-dose FBP images with 100 kVp tube voltage, a noise index of 23.5, and a 0.625 mm slice thickness, (2) low-dose MBIR images with 100 kVp tube voltage, a noise index of 37.8, a 0.625 mm slice thickness, and MBIR-80% reconstruction, and (3) low-dose DLIR images with 100 kVp tube voltage, a noise index of 37.8, and a 0.625 mm slice thickness, and DLIR-high reconstruction. Noise power spectrum (NPS) was evaluated following the AAPM guidelines and then compared among the three sets of images.

### 2.2. Clinical Study on CCTA

#### 2.2.1. Study Population

**Figure 1:**
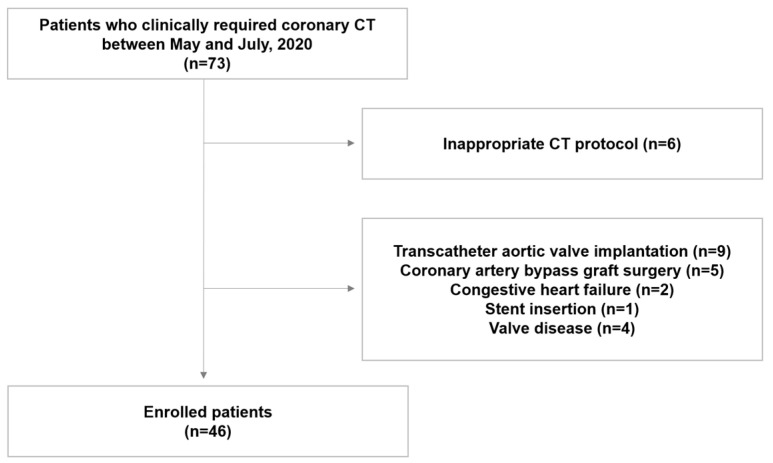
Flow chart of the study population.

This was a retrospective, observational study. Forty-six participants (23 men and 23 women; mean age 66.8 ± 14.5 years) who clinically required coronary CT were retrospectively enrolled in the study between May and July 2020. The exclusion criteria were different voltages of the CT protocol, such as 120 kVp or 80 kVp, patients who underwent coronary artery bypass graft surgery (CABG) or transcatheter aortic valve implantation (TAVI), insertion of a coronary stent, and known cardiac valve disease or congestive heart failure (Figure 1). Our institutional review board (Gangnam Severance Hospital IRB) approved this study, and the requirement for written informed consent was waived, due to the retrospective nature of the study. Patient records and information were anonymized and de-identified prior to the analysis.

#### 2.2.2. CCTA Protocol

All CCTA scans were performed using a 256-detector-row CT scanner (Revolution CT; GE Healthcare, Waukesha, WI, USA). Data acquisition was performed in the craniocaudal direction during a single breath hold at the end-inspiratory pause. The scanning range encompassed the heart, from the level of the carina to the diaphragm.

The scanning parameters were as follows: 100 kVp tube voltage, a noise index of 37.8, a 256 × 0.625 mm detector collimation with dynamic focal spot imaging, and a 280 ms gantry rotation time. Ioversol with an iodine concentration of 350 mg/mL (Optiray 350; Tyco Healthcare, Kantata, Toronto, ON, Canada) was administered intravenously through an antecubital 18-gauge catheter for 13 s, using a power injector (Dual Shot; Nemoto Kyorindo, Tokyo, Japan). The start time of data acquisition was determined using a real-time bolus tracking technique, and scans began 7 s after attaining a trigger threshold of 100 Hounsfield units (HU) in the descending aorta. The breath-hold maneuver was successfully performed in all the scans. The ECG signals were recorded simultaneously during each study (Table 1).

#### 2.2.3. CT Analysis

The CT images were reconstructed using FBP, MBIR-40%, MBIR-60%, MBIR-80%, DLIR-L, DLIR-M, and DLIR-H. The reconstruction parameters were as follows: a 0.625-mm slice thickness, a 0.625 mm increment, a 512 × 512 pixel image matrix, an XCC kernel, and a 15–23 cm field of view.

All images were reviewed and interpreted using AW Server 3.2 Ext. 1.0. The aortic attenuation values for all participants on axial CT images were measured using a round region of interest larger than 2.5 cm^2^ at the ascending aorta. Coronary artery attenuation values were measured using a round region of interest larger than 0.4 cm^2^ at the proximal right coronary artery (RCA), left main coronary artery (LM), left anterior descending artery (LAD), and left circumflex artery (LCx). Image noise was evaluated using CCTA based on one standard deviation of the attenuation value at the ascending aorta. The signal-to-noise ratio (SNR) was calculated as the vascular attenuation/image noise. The contrast-to-noise ratio (CNR) was calculated as [(attenuation of the vessel)—(attenuation of the adjacent perivascular fat)]/image noise. These parameters were compared for each reconstruction method.

Two experienced radiologists with more than 10 years of experience in cardiac CT independently conducted a qualitative assessment of the image quality of CCTA. The 18 coronary segment models and a four-point grading scale were used for qualitative evaluation: 1 = (poor/non-diagnostic), severe image degradation or discontinuation of vessel contour that prevented vessel lumen evaluation; 2 = (adequate), moderate image degradation that impeded vessel lumen evaluation; 3 = (good), minor image degradation that did not affect vessel lumen evaluation; and 4 = (excellent), no image degradation.

### 2.3. Statistical Analysis

Continuous variables are presented as mean ± standard deviation (SD). Categorical variables are expressed as frequencies or percentages. The distribution of data was checked using the Shapiro–Wilk test. A linear mixed model with a post-hoc test was used to evaluate the statistical significance of the differences in image noise, attenuation, SNR, CNR, and image quality score. A kappa statistic was used to evaluate the inter-reader agreement for the qualitative assessment of the CCTA image quality. A *p*-value or Bonferroni-adjusted *p*-value of <0.05 was considered statistically significant. Statistical analyses were performed using commercially available software, SPSS 23 Statistical Package for the Social Sciences (Chicago, IL, USA) or SAS, version 9.4 (SAS Institute Inc., Cary, NC, USA).

## 3. Results

### 3.1. Phantom Study

#### 3.1.1. Noise

**Figure 2:**
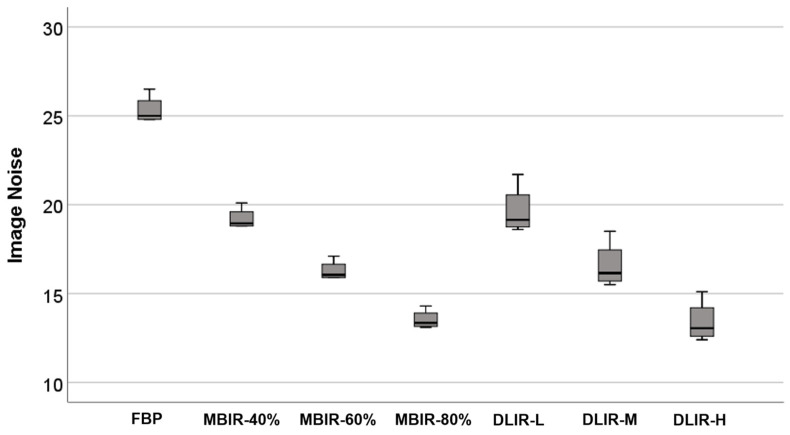
Image noise of the phantom study.

The noise value was calculated as follows: FBP was 25.3 ± 0.8; MBIR was 19.2 ± 0.6, 16.3 ± 0.6 and 13.5 ± 0.5 at 40%, 60%, and 80%, respectively; and DLIR was 19.7 ± 1.4, 16.6 ± 1.3 and 13.4 ± 1.2 at low, medium, and high, respectively. Compared with FBP, the noise reduction ratio of DLIR-H was 47.1%, and that of MBIR-80% was 46.6% (Figure 2).

#### 3.1.2. Noise Power Spectrum

**Figure 3:**
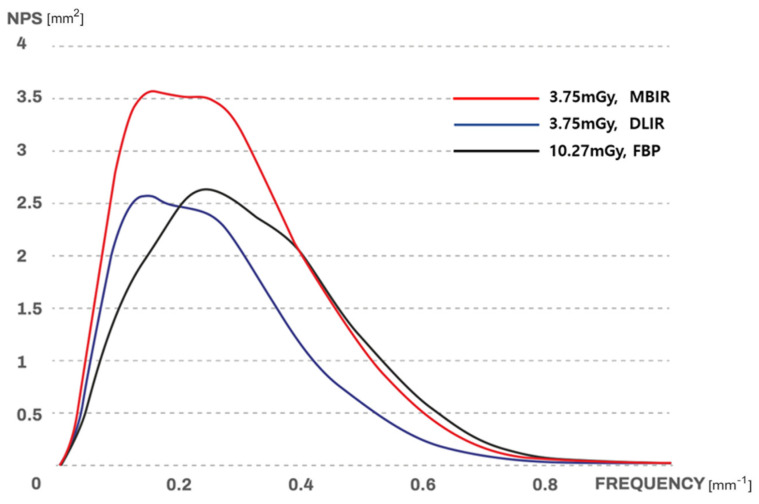
Noise power spectrum of the phantom study. The black line represents the noise power spectrum (NPS of an image obtained by FBP reconstruction of raw data obtained using a high radiation dose (10.27 mGy for a noise index of 23.5). The red line represents the NPS of an image obtained by MBIR-80% reconstruction of raw data obtained using a low radiation dose (3.75 mGy for noise index 37.8). The blue line represents the NPS of an image obtained by DLIR-H reconstruction of raw data obtained using a low radiation dose (3.75 mGy for noise index 37.8). The black and blue lines show similar shapes, but the red line is different.

For the high-dose FBP image, 10.27 mGy was used with a noise index of 23.5, while 3.75 mGy was used with a noise index of 37.8 for the low-dose MBIR or DLIR images. Figure 3 shows the noise power spectrum of the three sets of phantom images. The pattern of the noise power spectrum of the DLIR images was more similar to that of the high-dose FBP images than that of the MBIR images (Figure 3).

### 3.2. CCTA

#### 3.2.1. Demography

In this study, we examined 46 patients who clinically needed CCTA, and all procedures were performed without complications. The demographics of the study population are presented in Table 2. The mean heart rate was 74.0 ± 12.8, and the mean dose length product was 182.5 ± 120.4 mGy·cm.

#### 3.2.2. Results of the Quantitative Image Quality

**Figure 4:**
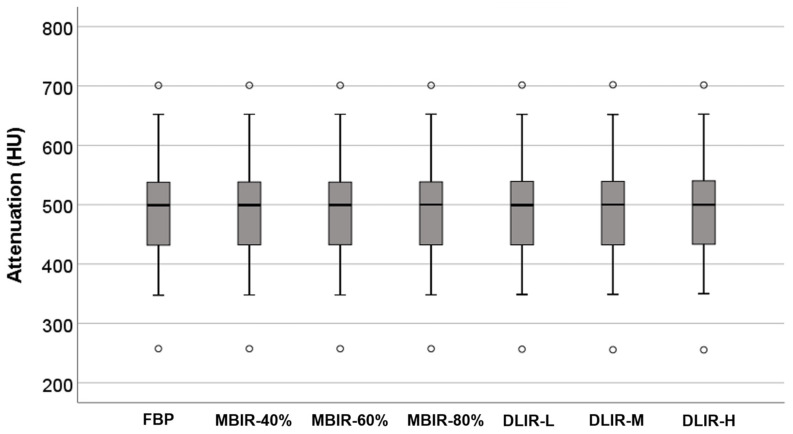
Attenuation of CCTA according to the reconstruction methods. Attenuation was measured in the ascending aorta. There are no significant differences in the images reconstructed by FBP, MBIR-40%, MBIR-60%, MBIR-80%, DLIR-L, DLIR-M, and DLIR-H.

The mean CT attenuation value of the aortic root on the CCTA with FBP was 489.5 ± 90.3. The attenuation measured at the ascending aorta did not differ significantly in each of the seven reconstructed images (all *p* > 0.05) (Figure 4).

**Figure 5:**
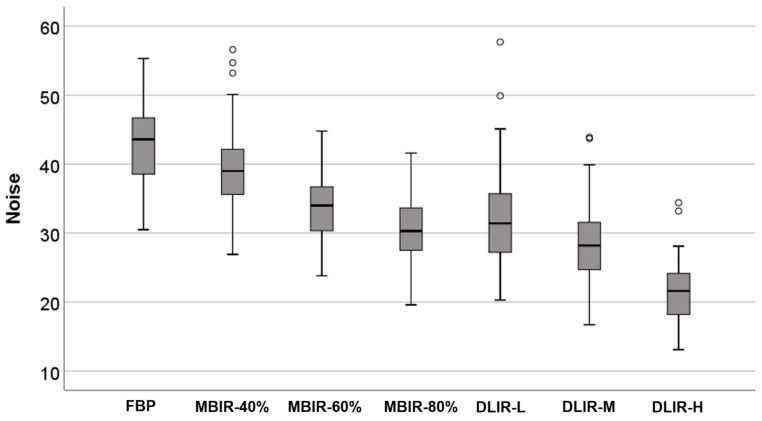
Image noise of CCTA according to the reconstruction methods. The noises of MBIR-80% and DLIR-H are lower than that of the FBP. Compared with FBP, the noise reduction ratio of DLIR-H is 49.6%, and that of MBIR-80% is 28.0%.

The image noise of DLIR-H was 21.4 ± 4.5, which was significantly lower than that of MBIR-80% (30.6 ± 4.5) and FBP (42.5 ± 5.7) (*p* < 0.05) (Figure 5). Compared with FBP, the noise reduction ratio of DLIR-H was 49.6%. The SNR and CNR of each coronary artery with DLIR-H were significantly higher than those of FBP and MBIR-80% (all *p* < 0.05) (Table 3).

#### 3.2.3. Results of the Qualitative Image Quality

**Figure 6:**
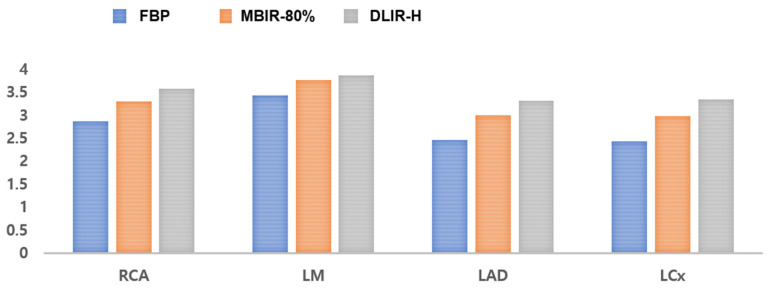
Qualitative image qualities according to the reconstruction methods.

In total, 595 segments with a diameter greater than 1.5 mm were evaluated on CCTAs with FBP, MBIR-80%, and DLIR-H. On CCTA with DLIR-H, 586 segments were scored as diagnostic (grades 2–4), with 292 (49.1%) graded as excellent, 239 (40.2%) graded as good, and 55 (9.2%) graded as adequate. Nine segments (1.5%) were considered non-diagnostic. On CCTA with MBIR-80%, 564 segments were scored as diagnostic (grades 2–4), with 184 (30.9%) graded as excellent, 262 (30.9%) graded as good, and 118 (19.8%) graded as adequate. Thirty-one segments (5.2%) were considered non-diagnostic. On CCTA with FBP, 522 segments were scored as diagnostic (grades 2–4), with 69 (11.6%) graded as excellent, 238 (40%) graded as good, and 215 (36.1%) graded as adequate. Seventy-three segments (12.3%) were considered non-diagnostic (Table 4). The mean image quality scores of the segment-based analysis are shown in Table 5 and Figure 6. The inter-reader agreement for the qualitative assessment of the CCTA image quality was 0.743 for FBP, 0.816 for MBIR-80%, and 0.809 for DLIR-H.

## 4. Discussion

This study aimed to evaluate the feasibility of DLIR on CCTA. The results show that the DLIR algorithm is feasible and yields a better image quality than the model-based iterative reconstruction algorithm in CCTA.

CCTA is a non-invasive tool for the evaluation of coronary artery diseases, such as non-stenotic atheroma and stenosis. However, patients who undergo CCTA cannot avoid the radiation exposure and toxicity of contrast, which is a critical limitation of CCTA [13]. One way to reduce the harm caused by CCTA is to improve the reconstruction method.

FBP, which has been used for the longest time, is an efficient program that is reconstructed in near real-time while scanning the target. However, it requires a relatively high dose of radiation to acquire a fine-quality image owing to image noise [14,15]. Many noise reduction techniques have been developed to reduce the radiation dose while maintaining image quality; IR is one of them, which is the most popular [16]. First-generation IRs, such as IRIS (Iterative Reconstruction in Image Space, Siemens Healthcare, Erlangen, Germany) and AIDR (Adaptive Iterative Dose Reduction, Toshiba Medical Systems Corp., Otawara, Japan), repeatedly apply algorithms only to image domains, sequentially removing noise and reconfiguring objects [17,18,19]. Hybrid IR, such as SAFIRE (Sinogram Affirmed Iterative Reconstruction, Siemens Healthcare), ASiR (Adaptive Statistical Iterative Reconstruction, GE Healthcare, Waukesha, WI, USA), iDose (Philips Healthcare, Andover, MA, USA), and AIDR 3D (Adaptive Iterative Dose Reduction three dimensional, Toshiba), which combines both analytical and iterative methods, refers to an algorithm that creates an initial image using an analytical reconstruction method in the raw data domain and then reconstructs it repeatedly to optimize image characteristics, such as eliminating noise in the image domain [20,21]. Model-based IRs, such as Veo (GE Healthcare), ASiR-V (GE Healthcare), IMR (iterative model-based reconstruction, Philips Healthcare), and FIRST (forward-projected model-based IR solution, Canon Medical System, Otawara, Japan), use geometric and statistical models, such as focus, voxel, and detector size, to correct the initial calculation value of a video and utilize the physical model and the characteristics of medical images. In other words, it refers to IR, which considers video acquisition processes, statistical models, and even system geometry models [22]. IR algorithms provide the ability to reduce noise in low-radiation dose datasets, but they also have disadvantages, such as edge-definition artifacts [23] and the degrading of diagnostic performance by blotchy noise texture in low-dose settings [16,24]. DLIR based on deep neural networks (DNNs) can handle millions of parameters using mathematical equations. The DNN is trained by repeating the process of inputting a low-dose sonogram and comparing the output image to a high-dose version of the same data until there is accuracy between the output image and the ground truth image. One of the DLIR programs, TrueFidelity™ (GE Healthcare, TF), uses an input image reconstructed by FBP; therefore, the image reconstructed by TF has a more natural texture than the image reconstructed by IR. Our phantom study results showed a similar pattern of NPS to that of FBP with high-dose radiation and that of DLIR with low-dose radiation and support the strength of DLIR with natural image texture. DLIR has the additional strength of improving the reconstructed image quality to reduce noise. Greffier et al. [25] showed that DLIR reduced noise and improved spatial resolution and detectability in a phantom study. Our phantom study showed comparable results in that the noise reduction ratio was similar in DLIR-H and IR-80%, and noises with both were significantly lower than that of FBP.

**Figure 7:**
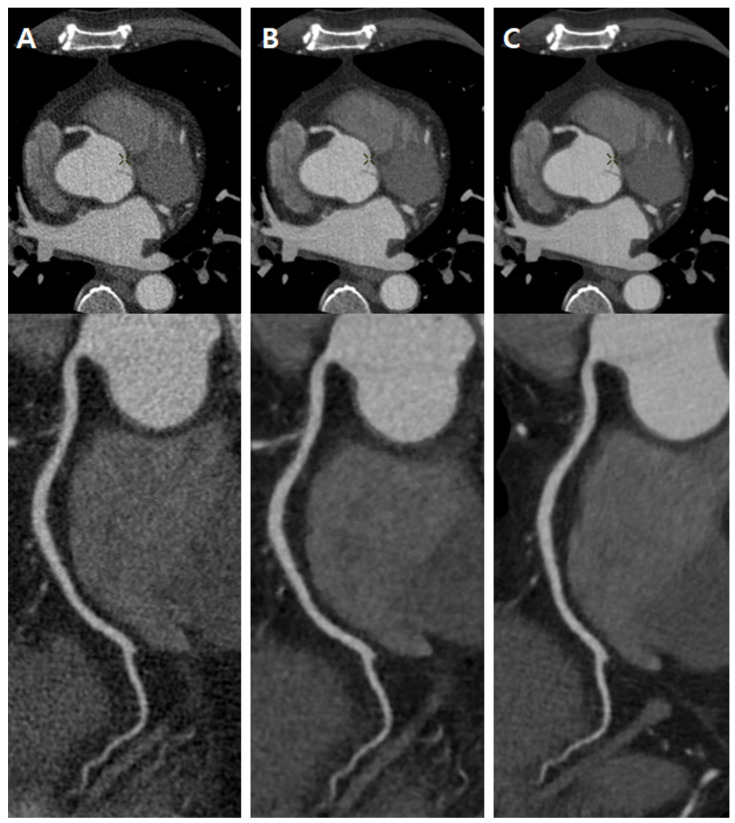
Representative CCTA images. CCTA of a 59-year-old male (BMI 25.25 kg/m^2^, heart rate 71 BPM, radiation dose 202.52 mGy·cm). (**A**) Reconstructed to FBP. (**B**) Reconstructed to MBIR-80%; the noise is reduced compared to the FBP image, but the noise particle size is increased, and the margin is relatively unclear. (**C**) Reconstructed to DLIR-H, it has a lower noise level than FBP, and the image texture appears natural compared to the MBIR image.

In the clinical application of DLIR, several studies evaluated the image quality of DLIR and compared it with FBP and IR in clinical settings. Park et al. [26] showed that TF-H was superior to ASiR-V in terms of image noise and sharpness on lower-extremity CT angiography. Heinrich et al. [27] showed that DLIR substantially improved objective and subjective image quality compared with iterative reconstruction in CT angiography of the aorta. Especially in CCTA, some studies have shown that DLIR significantly reduced noise with superior image quality [28,29]. Our study showed similar results: image noise on DLIR-H was significantly lower than that on IR and FBP, and the quality of the image on DLIR-H was better than that on MBIR-80% and FBP (Figure 7, Supplementary Figure S1).

Not all studies on DLIR have shown that it is superior to IR. Some studies have shown that there is no significant difference in low-contrast spatial resolution between IR and DLIR [30,31]. Jensen et al. [32] showed that medium- and high-strength DLIR were not significantly different from ASiR-V in terms of lesion diagnostic confidence or lesion conspicuity on abdominal CT. Furthermore, slight blurring was observed for lesions smaller than 5 mm and tiny vessels on some DLIR-H images. Our phantom study showed no significant difference in the noise reduction ratio of images reconstructed to DLIR-H and MBIR-80%. In CCTA, there was no statistically significant difference between DLIR-H and MBIR-80% in the qualitative image score of LM. 

Our study had a few limitations. First, the study population was relatively small and registered at a single institution. Second, the qualitative evaluation of CCTA was based on visual assessment alone, and we did not evaluate diagnostic accuracy [33]. Third, there were no direct comparisons between images derived from conventional and low radiation doses. However, our study included NPS and phantom studies for comparison of FBP, MBIR, and DLIR, and reliable results showed statistically significant differences between the reconstructed images. Further studies using DLIR in larger populations are expected to reduce the radiation dose while preserving the image quality of CCTA images.

## 5. Conclusions

The image texture of a phantom with low-dose DLIR-H was similar to that of high-dose FBP. In CCTA, DLIR-H showed better image noise reduction than MBIR and FBP. The DLIR algorithm is feasible and has better image quality than the FBP or MBIR algorithms on CCTA.

## Figures and Tables

**Table 1:** CT protocol.

Scan Type	1-Beat Cardiac Axial
Rotation type (s)	0.28
Slice (mm)	0.625
Tube voltage (kVp)	100
Noise index	37.8
Tube current (mA)	Care dose
Coverage (cm)	16
ECG gating	Auto
Bolus tracking	Ascending aorta
**Contrast protocol**	
Iodine concentration (mg/mL)	350
Total contrast dosage (mL/kg)	0.8
Injection time (s)	13
Injection rate (mL/s)	Total dosage/13
Saline flush	4-5 cc/s, Triphasic

**Table 2:** Demographic and basic characteristics of the patients.

Characteristics	Values
Number of participants	46
Age (years)	66.8 ± 14.5
Male:Female	23:23
Height (cm)	163.0 ± 8.7
Body weight (kg)	64.1 ± 10.5
Body mass index (kg/$m^{2}$)	24.1 ± 3.3
Average heart rate (beats/min)	74.0 ± 12.8
Dose length product (mGy·cm)	182.5 ± 120.4

**Table 3:** Results of the quantitative study.

	FBP	MBIR-40%	MBIR-60%	MBIR-80%	DLIR-L	DLIR-M	DLIR-H
**Attenuation**	489.5 ± 90.3	489.7 ± 90.3	489.7 ± 90.3	489.8 ± 90.3	489.7 ± 90.4	489.8 ± 90.5	490.1 ± 90.4
**Noise**	42.5 ± 5.7	39.5 ± 6.3	33.9 ± 4.5	30.6 ± 4.5	32.5 ± 7.1	28.6 ± 5.8	21.4 ± 4.5
**SNR**							
RCA	10.5 ± 2.4	11.3 ± 2.7	12.9 ± 2.6	14.3 ± 2.9	14.2 ± 3.5	16.0 ± 4.0	21.5 ± 5.4
LM	11.1 ± 2.6	11.9 ± 2.7	13.7 ± 2.6	15.2 ± 2.8	15.1 ± 3.8	17.1 ± 4.2	22.9 ± 5.6
LAD	10.3 ± 2.3	11.2 ± 2.7	12.8 ± 2.6	14.1 ± 2.9	14.1 ± 3.5	15.9 ± 4.0	21.3 ± 5.4
LCx	10.7 ± 2.6	11.5 ± 2.4	13.1 ± 2.6	14.5 ± 2.7	14.5 ± 3.6	16.3 ± 3.8	21.9 ± 5.5
**CNR**							
RCA	12.6 ± 2.5	13.7 ± 2.9	15.6 ± 2.6	17.2 ± 2.9	17.1 ± 3.9	19.3 ± 4.3	25.9 ± 6.0
LM	13.1 ± 2.8	14.1 ± 3.1	16.2 ± 2.9	17.9 ± 3.1	17.8 ± 4.4	20.1 ± 4.8	27.0 ± 6.5
LAD	12.6 ± 2.5	13.6 ± 3.1	15.6 ± 2.9	17.3 ± 3.2	17.2 ± 4.1	19.4 ± 4.7	25.9 ± 6.2
LCx	12.9 ± 2.9	13.8 ± 2.8	15.8 ± 2.8	17.4 ± 3.0	17.4 ± 4.2	19.5 ± 4.4	26.2 ± 6.1

RCA, right coronary artery; LM, left main coronary artery; LAD, left anterior descending artery; LCx, left circumflex artery; MBIR, model-based iterative reconstruction; DLIR, deep-learning-based image reconstruction; SNR, signal-to-noise ratio; CNR, contrast-to-noise ratio.

**Table 4:** Table regarding the qualitative study: Number of segments for each.

	FBP	MBIR-80%	DLIR-H
**Grade 1** (Poor)	73 (12.3%)	31 (5.2%)	9 (1.5%)
**Grade 2** (Adequate)	215 (36.1%)	118 (19.8%)	55 (9.2%)
**Grade 3** (Good)	238 (40%)	262 (44.0%)	239 (40.2%)
**Grade 4** (Excellent)	69 (11.6%)	184 (30.9%)	292 (49.1%)

FBP, filtered-back projection; MBIR, model-based iterative reconstruction; DLIR, deep-learning-based image reconstruction.

**Table 5:** Table regarding the qualitative study: Mean value per vessel analysis.

	FBP	MBIR-80%	DLIR-H	*p*-Value
RCA	2.86 *	3.30 *	3.57	<0.001
LM	3.43 *	3.76	3.86	<0.001
LAD	2.46 *	3.00 *	3.31	<0.001
LCx	2.43 *	2.98 *	3.34	<0.001

RCA, right coronary artery; LM, left main coronary artery; LAD, left anterior descending artery; LCx, left circumflex artery; MBIR, model-based iterative reconstruction; DLIR, deep-learning-based image reconstruction; * indicates post hoc *p* < 0.05 compared to DLIR-H.

## Data Availability

All datasets generated or analyzed during the current study are available from the corresponding author on reasonable request.

## Supplementary Materials

The following supporting information can be downloaded at: https://www.mdpi.com/article/10.3390/jcm12103501/s1, Figure S1: Representative CCTA images with 7-different reconstruction methods.
