# Supplementary material for: Clinical Feasibility of Deep Learning-Based Image Reconstruction on Coronary Computed Tomography Angiography
Source: J Clin Med. 2023 May 16;12(10):3501. doi: 10.3390/jcm12103501 (PMC10219179; doi:10.3390/jcm12103501)
Supplement: Supplementary file 1 [file jcm-12-03501-s001.zip › jcm-2321961-supplementary.pdf]

Supplementary Figure

Figure S1. Representative CCTA images with 7-different reconstruction methods

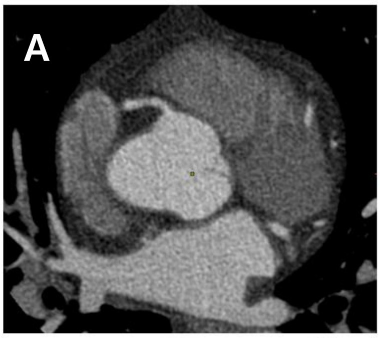

59/M

178cm, 80kg, BMI: 25.25kg/m^2

Heart rate: 71 bpm

100 kVp, DLP: 202.52 mGy*cm

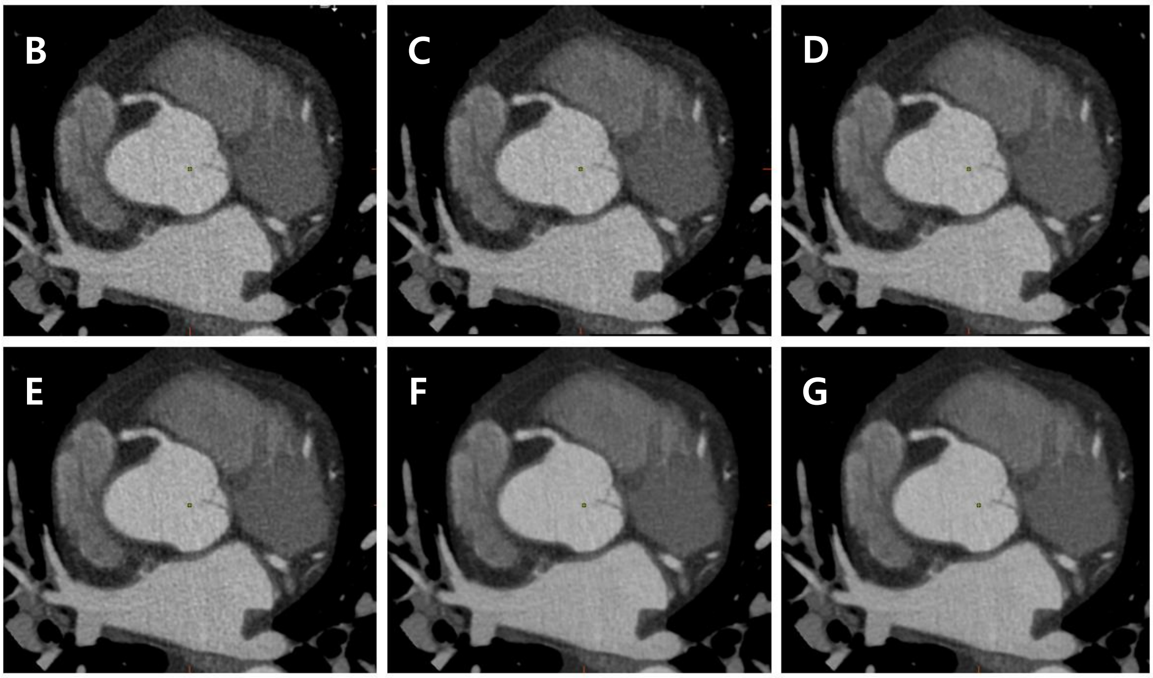

CCTA of a 59-year-old male.

(A) Reconstructed to FBP;

(B) Reconstructed to MBIR-40%; (C) Reconstructed to MBIR-60%; (D) Reconstructed to MBIR-80%;

(E) Reconstructed to DLIR-L; (F) Reconstructed to DLIR-M; (G) Reconstructed DLIR-H
